# Efficacy and Safety of Ziprasidone Injection vs Haloperidol Injection for Agitation in Patients with Acute Schizophrenia

**DOI:** 10.31083/AP40032

**Published:** 2025-04-28

**Authors:** Sufang Qi, Wenjie Li, Limin Yang, Guangwei Sun, Xinming Li, Xin Liu, Zhicheng Xue, Yue Zhang, Guanglei Xun

**Affiliations:** ^1^Shandong Mental Health Center, Shandong University, 250014 Jinan, Shandong, China

**Keywords:** S-methyl-dihydroziprasidone, N-(4-tert-butylbenzyl)haloperidol, schizophrenia, psychomotor agitation, mania

## Abstract

**Objective:**

Agitation represents a serious and prevalent symptomatology within acute schizophrenia. This study aims to conduct a nuanced comparison of the efficacy and safety profiles of intramuscular (IM) ziprasidone versus IM haloperidol in the management of agitation among patients with acute schizophrenia.

**Methods:**

This investigation was structured as a randomized, 3-day study, utilizing flexible dosing strategies. It included 69 patients diagnosed with schizophrenia, who were randomly allocated to receive either IM ziprasidone (n = 35, 20 to 40 mg/day) or IM haloperidol (n = 34, 5 to 10 mg/day). The primary endpoints included comparative analyses of the change in Positive and Negative Syndrome Scale (PANSS) total scores and Positive and Negative Syndrome Scale Excited Component (PANSS-EC) scores from baseline to study completion across the two groups.

**Results:**

At baseline, there were no significant differences between the IM ziprasidone and haloperidol groups. Both treatments led to significant reductions in PANSS-EC total scores (haloperidol, *p* = 0.001; ziprasidone, *p* = 0.001) and PANSS total scores (haloperidol, *p* = 0.001; ziprasidone, *p* = 0.001) from baseline to study endpoint. Nevertheless, no significant difference was observed between the two groups in terms of changes in PANSS-EC scores (*p* = 0.312) and PANSS total scores (*p* = 0.159) from baseline to endpoint. The haloperidol group exhibited a higher incidence of adverse events compared with the ziprasidone group, reaching statistical significance (*p* = 0.027).

**Conclusions:**

Our findings indicate that both medications are equally effective in controlling agitation symptoms. However, ziprasidone exhibited superior characteristics in safety and tolerability, particularly in reducing the incidence of extrapyramidal symptoms.

**Clinical Trial Registration:**

The study was registered at https://www.chictr.org.cn/showproj.html?proj=246996, registration number: ChiCTR2500100002, date of registration: 1 April 2025.

## Main Points

1. The research demonstrated that both intramuscular ziprasidone and haloperidol are equally effective in reducing agitation symptoms in acute schizophrenia patients, marking them as viable options for immediate management of severe agitation.

2. Ziprasidone was associated with fewer side effects, particularly lower rates of extrapyramidal symptoms, and required less use of anticholinergic medication compared with haloperidol, indicating a more favorable safety and tolerability profile.

3. These findings suggest that ziprasidone could be considered a preferable option for the fast management of agitation in schizophrenia due to its safety advantages, thereby informing clinical decision-making in selecting antipsychotic medications for acute episodes.

## 1. Introduction

Schizophrenia, a chronic condition, often manifests agitation through disruptive and violent behavior during its acute phases [1]. Investigative reports on aggressive behaviors in hospitalized patients with schizophrenia indicate an incidence rate fluctuating between 9.1% and 49.6%, averaging 28.0% [2]. This phenomenon contributes to up to 2 million emergency room visits annually in the USA, with approximately 21% attributed to schizophrenia-related crises [3]. Prompt intervention for agitation is, therefore, an integral component of effective schizophrenia management. Addressing agitation aims to ensure the individual’s safety while assisting patients in managing their emotions and maintaining behavioral control. Research into the pathophysiological underpinnings of agitation has elucidated disruptions in the noradrenergic, dopaminergic, gamma-aminobutyric, and serotonergic systems as contributing factors [4].

The pharmacological management of agitation in schizophrenia primarily involves the use of both typical and atypical antipsychotics, as well as benzodiazepines [5]. While oral and intramuscular (IM) administration of benzodiazepines, such as lorazepam and diazepam, offers effective sedation, they risk inducing excessive sedation and respiratory depression without addressing the underlying psychiatric symptoms [6]. Conversely, IM antipsychotics, notably IM haloperidol, are preferred for acute agitation management due to their rapid onset, despite the potential for extrapyramidal symptoms (EPS) [7, 8]. Parenteral atypical antipsychotic agents, heralded by ziprasidone’s availability in injectable form, have demonstrated efficacy within 15–30 minutes of administration while maintaining a favorable profile with lower incidences of EPS and oversedation [9]. However, they are not without risks, namely the potential for arrhythmias and corrected QT interval (QTc) [10] prolongation, concerns that second-generation antipsychotics (SGAs) also share to a lesser extent.

Faced with the critical need for swift symptom management amid concerns over adverse reactions, this study was launched to compare the safety and efficacy of IM ziprasidone and IM haloperidol in managing agitation among hospitalized acute schizophrenia patients in China. This research endeavors to provide clinical insights essential for navigating the complexities of pharmacological interventions in acute psychiatric care.

## 2. Methods

### 2.1 Samples

This study was conducted at the Shandong Mental Health Center (Jinan, China) from January 2023 to September 2024. This research included the participation of Chinese individuals, both male and female, aged from 18 to 65 years, who met the criteria for schizophrenia as outlined by the International Statistical Classification of Diseases and Related Health Problems, 10th Revision (ICD-10), under the category of F20.X. The inclusion was specific to those in the acute phase of schizophrenia, with the stipulation that they could be administered IM medication for a minimum of 3 days based on the clinical judgement of the conducting researchers. Criteria for entry into the study included a Positive and Negative Syndrome Scale (PANSS) overall score of 70 or higher, a Positive and Positive and Negative Syndrome Scale Excited Component (PANSS-EC) score of 15 or higher, along with an Agitation-Calmness Evaluation Scale (ACES) score not exceeding 3.

### 2.2 Exclusion Criteria

Exclusion criteria: (1) alcohol and/or substance abuse; (2) a previous diagnosis of another mental disease; (3) thyroid illness, gout, kidney disease, major organic brain disease, immune disease, diabetes, etc.; (4) an infection in the previous 4 weeks or use of anti-inflammatory drugs, antibiotics, or glucocorticoids; (5) breastfeeding or pregnant; (6) regular use of psychiatric drugs in the past 2 weeks; (7) validated clinical significant abnormal laboratory values; (8) QTc prolongation or a prodrug QTc over or equal to 450 milliseconds; (9) known allergy to ziprasidone or haloperidol; and (10) those who were treated with electroconvulsive therapy during the previous 4 weeks. All eligible patients underwent a psychiatric assessment to determine if they satisfied the inclusion or exclusion criteria.

### 2.3 Grouping and Treatment

The subjects were randomly divided into equally sized groups to receive either ziprasidone (20 to 40 mg/d) (Jiangsu Nhwa Pharmaceutical Co., Ltd., lot No.: XN05AEQ010B001010101435, Xuzhou, Jiangsu, China) or haloperidol (5 to 10 mg/d) (Shandong Lukang Pharmaceutical Group Set limited liability company, lot No.: XN05ADF085B002020104166, Taian, Shandong, China) for 72 hours (3 days). This research used an unfixed-dose design; the dose range for ziprasidone reached 20 to 40 mg/d, and for haloperidol, 5 to 10 mg/d. These doses were regulated in accordance with the investigators’ clinical decisions. Research visits included a baseline visit, and at 72 hours after the first-dose time points.

Psychiatric evaluation utilized an array of scales: the PANSS, the PANSS-EC, the ACES and Barnes Akathisia Rating Scale (BARS) [11]. Scale evaluation is based on the performance of the patients on the day of testing. The PANSS-EC includes five items, with each item rating between 1 and 7, culminating in a total score range of 5 to 35 points. This scale is specifically designed to assess the intensity of hostile and restive symptoms. The PANSS features 30 items, rated similarly from 1 to 7 points, resulting in an overall score range from 30 to 210, and is employed to appraise the severity of psychotic manifestations.

Additionally, multiple scales were employed to assess the side effects linked to pharmacological interventions. These include the Rating Scale for Extrapyramidal Side Effects (RSESE), consisting of 10 items with scores ranging from 1 to 5 on each, the Barnes Akathisia Scale (BAS) comprising four items, each rated 0 to 3 for the initial three items and 0 to 5 for the last, resulting in a total possible score of 0 to 14. The Treatment Emergent Symptoms Scale (TESS) has 36 items, with each item graded on severity (0–4 points), the relationship to medication (0–4 points), and actions taken as a response to symptoms (0–6 points).

Laboratory examinations were also performed, such as complete blood cell counts, and electrocardiography at baseline and study endpoint, with the intention of evaluating the patients’ physical condition.

The primary measure of effectiveness was to compare the mean alterations in the PANSS total and PANSS-EC scores from the study’s onset to its completion between the groups receiving IM ziprasidone and haloperidol. The principal measure for analysis was the rate of reduction in the PANSS-EC and PANSS scores, calculated as [(baseline score – endpoint score)/baseline score] × 100%.

Secondary measures of efficacy involved comparisons of the differences in the changes observed in ACES and BARS scores between the two treatment cohorts. Safety outcomes were primarily focused on contrasting the frequency of anticholinergic medication use and the occurrence of adverse effects between the ziprasidone and haloperidol groups.

Blinding was maintained for subjects and all members of the research team regarding treatment allocation, except for the onsite rater. The evaluators responsible for assessing scale scores at each site visit were blinded to the treatment conditions; they also underwent standardized training before the commencement of the study to ensure consistency in evaluation. The same evaluator at each site conducted all assessments for each participant to maintain uniformity in ratings.

The usage of the other antipsychotics and parenteral benzodiazepines, or the prophylactic treatment of extrapyramidal reactions (EPS) with anticholinergics or propranolol was not permitted during the study period. Benzodiazepines were allowed for patients with insomnia. Anticholinergics or propranolol were allowed to be used in patients with EPS.

### 2.4 Safety

Tolerability and safety assessments were carried out initially and again at the 72-hour mark, encompassing electrocardiograms, complete blood counts, and evaluations for any side effects. QT intervals were corrected for heart rate using QTc. Evaluations for EPS were conducted using the BAS and the RSESE both at the starting point and after 72 hours.

We reported side effect which took place during this research and validated the precision of the clinical report on the grounds of the associated scale evaluation and physical test indexes. When assessing the adverse effects of EPS upon the RSESE scale and Barnes scale, doctors frequently recommend medications like anticholinergics. Through the clinical reporting of adverse effects, insomnia, excessive sedation, and dizziness were detected. While the BARS index was less than 3, those subjects were identified as excessive sedation patients.

### 2.5 Statistical Methods

To rigorously analyze the collected data, we first conducted a preliminary examination to assess the distribution of the dataset. Recognizing that not all variables followed a normal distribution, we deployed descriptive statistics for a comprehensive summary. For variables that were normally distributed, we used the mean ± standard deviation (SD) to outline their central tendency and variability. Conversely, for those variables not aligning with normal distribution, we provided median and interquartile range (Q1, Q3) as representations, thus offering insight into the data’s distribution and spread. To delineate differences between groups where the data followed normal distribution, the independent samples *t*-test was applied, an addition aimed at addressing the oversight in initial specifications of our analytic approach. For comparative analysis between groups in instances of non-normal data distribution, we utilized the Mann Whitney U test. Categorical variables reported as n (%) underwent analysis through either the Pearson Chi-squared test or Fisher’s exact test, depending on suitability to discern any notable associations or divergences. Since the proportion of cells with theoretical frequency less than 5 was greater than 20%, and some cells even had 0, we used Fisher-Freeman-Halton test to compare the marital status of the two groups, and the same was used for drug side effects. An integral facet of our safety assessment—the frequency of side effects—was meticulously documented and subjected to statistical evaluation to establish any significant variations across the treatment cohorts. All statistical tests prescribed to a significance threshold established at α = 0.05, denoting statistical significance. This decision aligns with standard practices in clinical research, facilitating consistency in our interpretation of the findings. The comprehensive data analysis was carried out using SPSS version 25.0 (SPSS Inc., Armonk, NY, USA).

## 3. Results

### 3.1 Patient Demographic Data and Clinical Characteristics at Baseline

Seventy patients were screened, and 1 patient was excluded before grouping who had unstable medical conditions(=1). All 69 patients were treated with the study drugs, 35 patients with ziprasidone and 34 patients with haloperidol, and they underwent baseline assessment. None of them left the study before study completion. Over the course of the 72-hour timeframe, the most commonly administered dosage was 40 mg for ziprasidone, achieved by 30 (85.7%) of the subjects, and 5 mg for haloperidol, achieved by 30 (88.23%) of the participants.

The study cohort comprised individuals diagnosed with schizophrenia, presenting moderate to severe psychotic symptoms, with an average age of 31.5 years. The mean duration of schizophrenia among the participants was approximately 4 years. At the initiation of the study, there were no statistically significant differences in clinical and demographic characteristics, nor in electrocardiogram and hematological test results (except for white blood cell count, *p* = 0.018) between the two treatment groups (Table 1).

**Table 1.** **Patient demographic data and clinical characteristics at baseline**.

		Haloperidol (n = 34)	Ziprasidone (n = 35)	t/χ^2^/Z	Df	*p*
Age, mean (SD), y		32.35 ± 11.93	30.80 ± 10.14	0.583	67	0.562^a^
Gender: male, n (%)		18 (53%)	23 (66%)	1.167	1	0.280^b^
Marriage, n (%)						
	Unmarried	22 (64.71%)	22 (62.86%)	3.054	3	0.359^c^
	Married	11 (32.35%)	8 (22.86%)			
	Divorced	1 (2.94%)	4 (11.43%)			
	Widowed	0 (0.00%)	1 (2.86%)			
Duration of schizophrenia, median (Q1, Q3)		56.50 (32.0, 154.5)	47.00 (21.0, 128.0)	–0.636		0.525^d^
PANSS mean (SD)		92.71 ± 10.52	94.34 ± 8.43	–0.714	67	0.478^a^
PANSS-EC, mean (SD)		20.35 ± 2.19	20.00 ± 3.07	0.552	61.52	0.583^a^
ACES, mean (SD)		2.41 ± 0.61	2.34 ± 0.76	0.413	67	0.681^a^
BRAS, mean (SD)		5.41 ± 0.78	5.29 ± 0.86	0.636	67	0.527^a^
White blood cell count, mean (SD)		7.66 ± 2.55	6.37 ± 1.84	2.415	67	0.018^a^
CRP, median (Q1, Q3)		0.60 (0.20, 1.78)	0.84 (0.30, 3.30)	1.336		0.181^d^
Heart rate, mean (SD)		89.06 ± 16.81	87.69 ± 15.98	0.348	67	0.729^a^
QTc interval of ECG, mean (SD)		425.91 ± 14.05	422.11 ± 22.11	0.854	57.85	0.397^a^

PANSS, Positive and Negative Syndrome Scale; PANSS-EC, Positive and Negative Syndrome Scale Excited Component; ACES, Agitation-Calmness Evaluation Scale; BARS, Barnes Akathisia Rating Scale; CRP, C-reactive protein; QTc, corrected QT interval; ECG, Electrocardiogram; Df, degree of freedom. ^a^The normal distribution data of age, scale score, etc., were analyzed by independent sample *t* tests; ^b^sex distribution difference between the two groups was tested using the Pearson Chi-squared test; ^c^Differences in marriages status were tested using the Fisher-Freeman-Halton test; ^d^Non-normal data were tested using the Mann Whitney U Test.

**Fig. 1.**
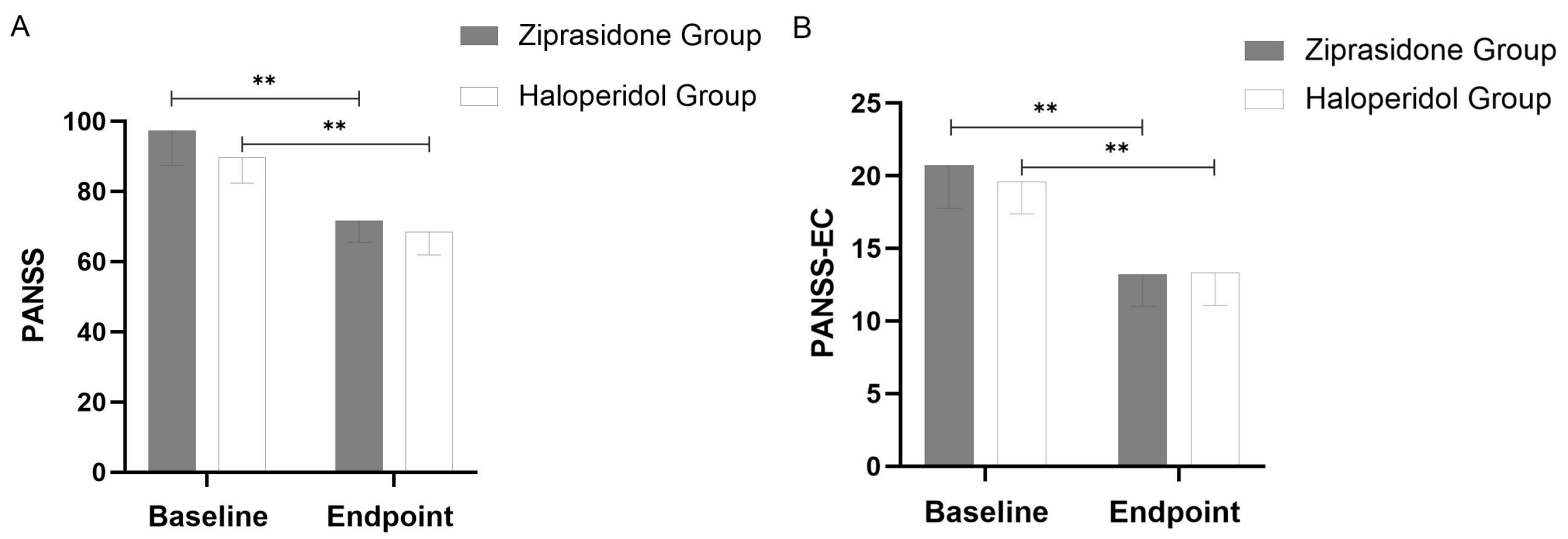
**Changes in PANSS Total Scores (A) and PANSS-EC Scores (B) from Baseline to Study Endpoint (using last observation carried forward methodology)**. Throughout the 72-hour period, a significant difference (*p* = 0.001) was observed between the two groups at all points. The error bars represent the standard deviation. **: *p* < 0.01.

Main assessment measures: PANSS-EC score and PANSS total score demonstrated a decreasing trend after 72 hours of treatment and was statistically significant (haloperidol, *p* = 0.001; ziprasidone, *p* = 0.001) (Fig. 1). There were no significant between-group distinctions in the subtraction rate of PANSS total score (*p* = 0.159) and PANSS-EC score (*p* = 0.312) (Table 2).

**Table 2.** **Comparison of changes in severity of psychotic symptoms from baseline to end of study between groups**.

	Haloperidol	Ziprasidone	t	Df	*p*
	(*n* = 34)	(*n* = 35)			
Reduction rate of PANSS total score	23.96 ± 4.01	25.58 ± 5.29	–1.423	67	0.159
Reduction rate of PANSS-EC score	33.53 ± 6.76	35.13 ± 6.33	–1.019	67	0.312

**Fig. 2.**
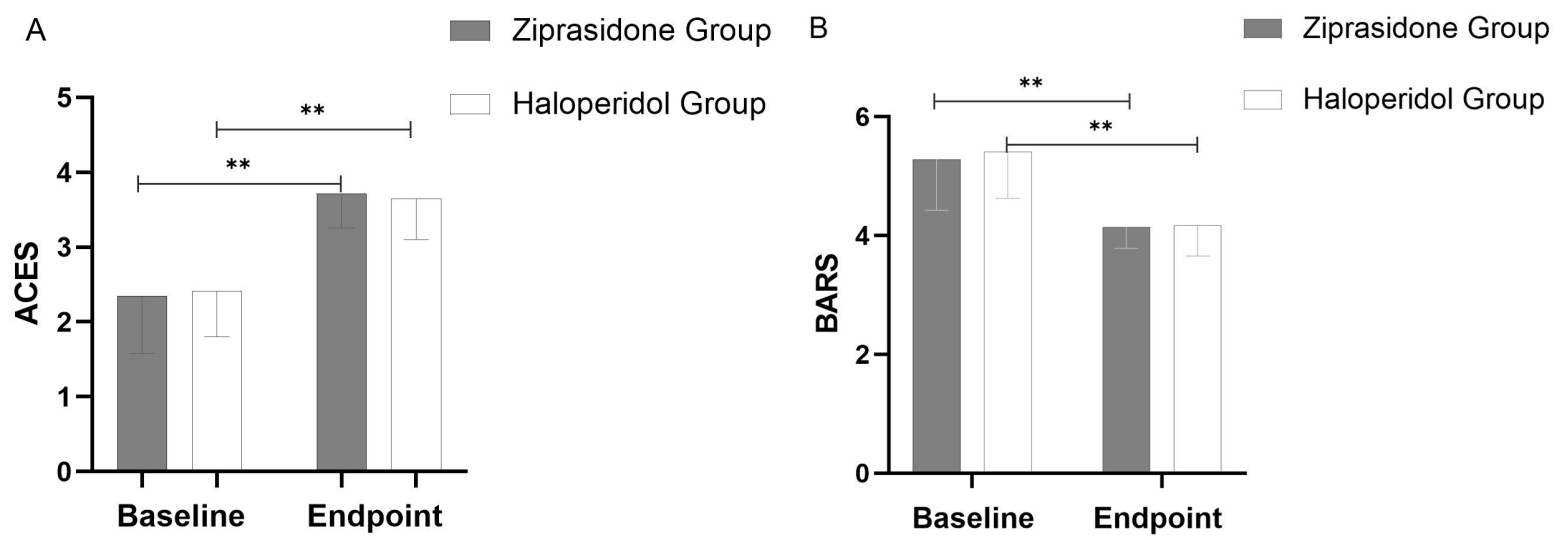
**Changes in ACES (A) and BARS (B) from Baseline to Study Endpoint (using last observation carried forward methodology)**. Throughout the 72-hour period, a significant difference (*p* = 0.001) was observed between the two groups at all points. The error bars represent the standard deviation. **: *p* < 0.01.

Secondary efficacy outcome: BARS score decreased significantly (*p* = 0.001) and ACES score increased significantly (*p* = 0.001) in the haloperidol and ziprasidone groups from baseline to the study endpoint (Fig. 2).

### 3.2 Safety Results

Side effects and the prescribed dosage were evaluated at baseline visit and at 72 hours. The presence of extrapyramidal syndrome in the two groups was detected. RSESE, BAS, and TESS were used for evaluating side effects that occurred during the study period.

Fewer patients suffered side effects in the ziprasidone group (n = 3, 8.57%) than in the haloperidol group (n = 10, 29.41%). The discrepancy between the groups was prominent (χ^2^ = 4.889, *p* = 0.027). No serious side effects or deaths occurred in either group. In the ziprasidone group, there was one case of extrapyramidal reactions and two cases of excessive sedation. In the haloperidol group, extrapyramidal reactions occurred in six cases, excessive sedation in three cases, and dizziness in one case. See Table 3. The use of anticholinergic drugs in the ziprasidone group was no more than that in the haloperidol group; the difference was not statistically significant (*p* = 0.055). See Table 3.

**Table 3.** **Adverse reactions in the ziprasidone and haloperidol groups**.

	Haloperidol	Ziprasidone	χ^2^	Df	*p*
	(*n* = 34)	(*n* = 35)			
Extrapyramidal symptoms (n)	6 (17.65%)	1 (2.86%)	-^a^	1	0.055
Excessive sedation (n)	3 (8.82%)	2 (5.71%)	-^a^	1	0.673
Dizziness (n)	1 (2.94%)	0	-^a^	1	0.493
Total (n)	10 (29.41%)	3 (8.57%)	4.889^b^	1	0.027

^a^The two groups were tested using the Fisher-Freeman-Halton test; ^b^The two groups were tested using the Pearson Chi-squared test.

## 4. Discussion

Within psychiatric hospitals in China, a variety of drug treatments are utilized to manage symptoms of agitation. Generally, there are two primary clinical strategies for addressing agitation. The first method employs antipsychotic medications, which work by reducing hyperactivity in dopaminergic neurons. The second approach makes use of benzodiazepines to suppress the activity of γ-aminobutyric acid (GABA) neurons, mainly to achieve a calming effect, although this method does not directly target the symptoms of psychosis. Currently, a significant amount of research, using haloperidol or a placebo as a control, is dedicated to assessing the safety and efficacy of new antipsychotics in managing schizophrenia [4, 7].

Patients with agitation symptoms are often described as disturbed and nervous, while physicians have found increased verbal or behavioral activity, irritability, uncooperation, and threatening attitudes, even leading to violence and aggression [4, 12, 13], and nearly half of patients who received medication intervention reported considerable overall satisfaction with agitation reduction and time to onset. At present, the pathogenesis of agitation is not fully understood. Benzodiazepines, typical antipsychotics, and atypical antipsychotics are widely employed for agitation, but their pharmacological mechanisms are different. The effects of benzodiazepines are due to their sedative effects [14, 15]. For this reason, a few psychiatrists believe that antipsychotics exert a more powerful sedative effect, and have a greater efficiency in lessening or mitigating agitation symptoms [16]. Our findings suggest that ziprasidone matches haloperidol in efficacy for the management of acute agitative episodes. As for the conventional treatment, antipsychotic drugs are generally considered to be slow in treating schizophrenia, but the antipsychotic effects of short-term injection of ziprasidone in this study are obvious, reminding us to pay attention to the influence of short-term IM injection on the overall treatment of the disease, and this effect might relate to the multi-receptor action of atypical drugs.

Patients in acute distress who are agitated, combative, or otherwise at risk for violent behavior are indicated mostly for treatments of rapid tranquilization. Under such circumstances, the actions of the patients may be harmful both for themselves and others around them, so immediate interventions are warranted, even within several minutes. Therefore, agitated behaviors are observed in many studies to assess the transformations in hours [7, 17]. To observe the rapid effect of the drug through the trial, the observation period was set at 3 days.

The primary evaluation metrics of the study revealed that, following 3 days of IM therapy, both the ziprasidone and haloperidol groups demonstrated significant decreases in PANSS-EC scores as well as overall PANSS scores. Notably, there was no significant difference in the rate of reduction between the two groups, indicating that ziprasidone was just as effective as haloperidol in diminishing psychotic and agitated symptoms—a result that aligns with our initial hypothesis. Secondary assessments further showed that participants in both the ziprasidone and haloperidol groups experienced significant improvements in ACES scores and a notable decrease in BARS scores. Moreover, significant enhancements in managing aggressive or disordered behaviors were observed in both groups, with ziprasidone proving to be equally efficacious as haloperidol in this regard. These findings are consistent with previous research on the application of ziprasidone and haloperidol in treating agitation in patients, reinforcing the established efficacy of these treatments [5, 9].

In terms of safety evaluation, it was found that the incidence rate of adverse effects in the haloperidol group greatly exceeded that in the ziprasidone group, which was consistent with previous studies [17, 18]. Nonetheless, the incidence of anticholinergic drug use was not statistically different between the ziprasidone and haloperidol groups.

Nevertheless, the study faced a few limitations. Initially, for ethical considerations, we did not establish a placebo control group. This omission possibly led to an inflated perception of SGA’s effectiveness on agitation symptoms, given that all subjects underwent potent antipsychotic treatment, potentially biasing patients and symptom evaluators towards expecting beneficial outcomes. Furthermore, while our research approach was earnest, the treatment preferences expressed by the patients might have subtly influenced the outcome measures. Moreover, the robustness of our findings was limited by the small sample size, which hampered our ability to detect differences between groups. This small cohort also reduced our capacity to evenly distribute potential confounding variables across the study. Additionally, conducting the research with a flexible dosing schedule means our results are not directly comparable to studies utilizing a fixed-dose regimen, which could also account for instances of inadequate dosing among some participants.

## 5. Conclusions

In summary, the primary purpose of this study was to provide clear guidance for clinicians in choosing between ziprasidone and haloperidol for the treatment of agitation symptoms in patients with acute schizophrenia, by comparing the safety and efficacy of these two medications. The results demonstrate that although both medications have similar effectiveness in symptom relief, ziprasidone shows better safety in minimizing adverse effects, especially extrapyramidal symptoms. This finding supports our expected goal that ziprasidone could be a safer and more suitable choice for treating agitation in schizophrenia patients compared with haloperidol. We encourage future research to delve further into this area to validate our findings and optimize treatment strategies, to enhance treatment outcomes and quality of life for patients.

## Availability of Data and Materials

All experimental data included in this study can be obtained by contacting the corresponding authors if needed.
